# Updates in the Management of Hereditary Periodic Fever Syndromes in Children

**DOI:** 10.7759/cureus.82284

**Published:** 2025-04-15

**Authors:** Saroj K Tripathy, Abhishek Kumar, Sarthak Das, Arvinder Wander, Soumi Kundu

**Affiliations:** 1 Pediatrics, All India Institute of Medical Sciences, Deoghar, Deoghar, IND; 2 Pediatrics, Shree Narayan Medical Institute and Hospital, Saharsa, IND; 3 Pediatrics, All India Institute of Medical Sciences, Bathinda, Bathinda, IND

**Keywords:** autoinflammatory diseases, biologics therapy, hereditary periodic fever syndromes, il-1, recurrent fever in children

## Abstract

Periodic fever syndrome is characterized by three or more febrile episodes in six months, each occurring at least seven days apart. Immune dysregulation in systemic autoinflammatory syndromes involves innate immunity (neutrophils, monocytes, and macrophages) and cytokines, mainly IL-1, with tumor necrosis factor (TNF), interferon alpha and beta, IL-2, IL-12, IL-18, and IL-23. Treatment includes colchicine, corticosteroids, immunosuppressants, and biologics. Prebiologic screening should be done to rule out tuberculosis, HIV, hepatitis B, and hepatitis C. Monitoring includes biomarkers of inflammation, disease activity score, looking for disease-specific organ involvement/complications, and treatment-related adverse drug reactions. There are research gaps in determining standardized treatment for the majority of autoinflammatory syndromes, duration of treatment, novel targets for treatment, and long-term prognosis.

## Introduction and background

Recurrent fever episodes in children are usually a result of infection, most likely caused by a viral etiology. Infection, autoimmune diseases, and malignancy are the causes of fever in children with pyrexia of unknown origin. Periodic fever syndrome is characterized by three or more febrile episodes in six months, each occurring at least seven days apart [1]. Periodic fever syndrome is a diagnosis of exclusion after ruling out other etiologies. Specific genetic mutations have been reported in different periodic fever syndromes. Hereditary periodic fever syndromes are also known as autoinflammatory syndromes. Unlike other rheumatic diseases, these syndromes are not associated with the involvement of adaptive immunity [2]. Immune dysregulation in systemic autoinflammatory syndromes involves innate immunity. Cells like neutrophils, macrophages, and natural killer (NK) cells are part of innate immunity. Autoinflammatory diseases involve cytokines, mainly IL-1, with tumor necrosis factor (TNF), interferon alpha and beta, IL-2, IL-12, IL-18, and IL-23 [3]. The treatment of hereditary periodic fever syndromes is not standardized and changes over time. Although traditionally nonsteroidal anti-inflammatory drugs (NSAIDs) and disease-modifying drugs are used for most of the autoimmune and autoinflammatory diseases, the treatment and outcome of these diseases have seen drastic changes in the last two decades with the development of targeted therapy and biologics. Based on existing and recent evidence, this review will briefly discuss the current management strategies and potential therapeutic options for the future of common hereditary periodic fever syndromes.

## Review

Familial Mediterranean fever (FMF)

It is the most common periodic fever with an autosomal recessive mode of inheritance and is associated with the MEFV gene mutation. The MEFV gene codes for pyrin, which is expressed in cells associated with the immune system, serosa, and synovium. The disease starts in childhood, and symptoms include fever lasting one to three days, serositis, and joint and skin involvement [4]. Of the FMF patients, 90% manifest before the third decade of life. In children, recurrent episodes of intermittent fever lasting 24 to 72 hours may be the sole manifestation, and other clinical features may evolve over time. Abdominal symptoms include pain in the abdomen, constipation, and rarely diarrhea. Systemic amyloidosis is the most dreaded complication of FMF, which develops in patients with persistently raised markers of inflammation even during periods between attacks [5]. Since the 1970s, colchicine has been the cornerstone of therapy in these patients, reducing acute attacks, preventing the frequency of episodes, and renal amyloidosis in the majority of cases. Colchicine acts by suppressing PYRIN activity, thereby inhibiting inflammation [6]. Adverse effects of colchicine include diarrhea, lactose intolerance, and, rarely, bone marrow suppression. In colchicine-unresponsive or intolerant children, IL-1 antagonists such as anakinra (IL-1 receptor antagonist) and canakinumab (anti-IL-1β monoclonal antibody) are used with a good success rate [7]. Canakinumab is usually preferred due to its long half-life. Anakinra may be preferred in children with conditions with high-risk infections, such as post transplantation, malignancy, or on dialysis. Rilonacept is a fusion protein consisting of the ligand-binding domains of the IL-1 receptor 1 and IL-1 receptor accessory protein linked to the Fc region of human IgG1 that neutralizes IL-1. Rilonacept showed promising results from small studies and has not yet been approved for use in FMF.

Tumor necrosis factor receptor-associated periodic syndrome (TRAPS)

TRAPS is inherited as an autosomal dominant trait with the involvement of the TNFRSF1A gene. Missense mutation of the cysteine residue causes structural alteration of the TNF receptor in leukocytes. Acute episodes usually last one to four weeks and are associated with characteristic eye and skin features. Acute episodes may be precipitated by stress, minor trauma, or exercise. Erythematous macules over the body and extremities, periorbital inflammation, conjunctivitis, arthralgia, and abdominal pain are common findings associated with this disease. Treatment of TRAPS is generally based on the severity of the illness. A short course of corticosteroids is usually effective in aborting acute attacks. Anti-TNF receptor agents, etanercept, and IL-1 inhibitors (anakinra, canakinumab) effectively control acute episodes of inflammation and prevent future attacks [8]. Infliximab has been found to exacerbate the disease in some cases. Ophthalmologic evaluation is warranted to rule out retinal infarcts or optic atrophy [9]. Colchicine is not effective in reducing the symptoms or preventing amyloidosis in TRAPS [10].

Cryopyrin-associated periodic fever syndromes (CAPS)

It consists of three overlapping diseases with autosomal dominant inheritance. IL-1 overproduction and gain-of-function mutation in the NLRP3 gene, which codes for cryopyrin, cause the inflammatory symptoms associated with CAPS [11]. The NLRP3 gene activates IL-1β, thereby causing inflammation. The three disorders are familial cold autoinflammatory syndrome (FCAS), Muckle-Wells syndrome (MWS), and neonatal-onset multisystem inflammatory disease (NOMID). FCAS presents with self-limiting recurrent episodes of fever, skin rash, and arthralgia on exposure to cold, and symptoms are more often observed in winter. Symptoms usually start from early infancy, associated with conjunctivitis, and last for less than 24 hours. MWS presents with myalgia, arthralgia, urticarial skin rash, and conjunctivitis, along with fever. Sensorineural hearing loss and amyloidosis are two significant complications observed in this condition. Among the CAPS, NOMID is the most severe phenotype associated with rash, bizarre ossification of joints, and chronic aseptic meningitis. Blindness and sensorineural hearing loss have been observed in many cases [12]. NSAIDs may provide symptomatic relief. Although high-dose corticosteroids and thalidomide have considerable efficacy, they are not commonly utilized due to undesirable effects. IL-1 receptor antagonist anakinra showed promise in treating CAPS. Anakinra has a half-life of four to six hours, necessitating daily injections, which cause injection site pain and swelling. Rilonacept (IL-1 Trap) was the first drug for CAPS that the US Food and Drug Administration licensed, especially for FCAS and MWS in adults and children aged 12 and over. Canakinumab, a monoclonal antibody that targets IL-1β, has been approved by the US Food and Drug Administration for use in adults and children over the age of four with FCAS and MWS phenotypes [13,14]. Common adverse effects include vertigo, nausea, diarrhea, rhinitis, and nasopharyngitis. Supportive therapy, like hearing aids, physiotherapy, orthopedic devices, and psychosocial support are important adjunct for quality care in these children.

Hyperimmunoglobulin D syndrome (HIDS)

HIDS with periodic fever syndrome is an autosomal recessive disease caused by a mutation of the MVK gene, leading to reduced mevalonate kinase enzymatic activity. The clinical spectrum is wide, ranging from milder forms in HIDS to severe forms in mevalonic aciduria. The clinical features usually start from infancy with recurrent fever episodes, skin rashes, joint pain, hepatosplenomegaly, and lymphadenopathy [15]. Skin rashes are erythematous, non-migratory, and sometimes painful rashes extending to palms and soles. In mevalonic aciduria, developmental delay, dysmorphic features, organomegaly, and hematologic abnormalities are observed. NSAIDs, colchicine, glucocorticoids, and intravenous immunoglobulin (IVIG) are used with varying rates of success. Evidence suggests that canakinumab is the first-line therapy in HIDS, with a good success rate in children with frequent episodes. Anti-TNF agents (etanercept) and tocilizumab may be tried in resistant cases [16].

Periodic fever, aphthous stomatitis, pharyngitis, and adenitis (PFAPA) syndrome

PFAPA syndrome is the most frequent syndrome linked with recurrent fever globally, except in areas of high endemicity for FMF. Fever episodes occur at almost constant intervals of three to six weeks. Cervical adenitis occurs but children with general lymphadenopathy with or without hepatosplenomegaly need evaluation for other etiologies. Aphthous stomatitis is found in up to 70% of cases and usually resolves in a week without any scarring. Unlike some other autoinflammatory disorders or chronic inflammatory conditions, inflammatory markers like serum amyloid A or C-reactive protein (CRP) (greater than 1.0 mg/dL) are elevated during the flares and return to undetectable levels between febrile episodes [17,18]. The treatment of PFAPA syndrome is still unclear. A single dose of prednisolone is highly effective in aborting an episode [19,20]. NSAIDs may be used to alleviate fever. Colchicine's long-term efficacy in treating FMF and the clinical and laboratory correlations between FMF and PFAPA serve as the main rationale for its usage as a preventative treatment for PFAPA. For PFAPA patients, colchicine may be a useful second-line treatment for minimizing recurrent fever episodes, especially if prednisone shortens the time between episodes. Cimetidine is an H2 antagonist that has also been found to be an effective preventive medication for PFAPA [21,22]. Individuals with PFAPA and low serum vitamin D levels may have higher CRP levels and more frequent febrile episodes. Supplementation with 400 IU/day of vitamin D reduced the duration and frequency of febrile episodes in these patients [23]. A prospective study from Slovakia found ketotifen to be effective in reducing the symptoms and frequency of attacks [24]. Adenotonsillectomy should be recommended to certain patients considering the self-limited course of PFAPA, which resolves before adolescence, and potential postoperative complications. It should be reserved for patients with severe recurrent febrile episodes lasting more than six months [25]. Anakinra is also effective in promptly resolving symptoms and recurrence.

Pyogenic arthritis, pyoderma gangrenosum, and acne (PAPA) syndrome

PAPA syndrome is an autosomal dominant disease associated with early childhood onset joint inflammation and skin lesions [26]. Sterile erosive arthritis and ulcerative skin lesions, like pyoderma gangrenosum, are typical findings observed in this disorder. With advancing age, dermatologic manifestations predominate. Pyogenic autoinflammatory diseases (PAID) include three distinct syndromes, namely, PAPA, Majeed syndrome (MS), and deficiency of IL-1 receptor antagonist (DIRA). PAPA is caused by a mutation of PSTPIP1 (proline serine-threonine phosphatase interacting protein), which interacts with pyrin to cause IL-1β activation [27]. Due to the rarity of this condition and the paucity of evidence, there is no standardized treatment for PAPA. TNF inhibitors and IL-1 antagonists have been used to control the skin and joint manifestations [28,29]. Methotrexate was useful in relieving joint symptoms in a few cases [30].

Deficiency of IL-1 receptor antagonist (DIRA)

It is an autosomal recessive disease with a mutation of the ILRN1 gene. It usually presents with fever, sterile osteolytic lesions, and pustular skin lesions. Mortality in untreated DIRA patients approaches 30%. Since IL-1 receptor antagonist deficiency is the primary cause of this disorder, lifelong supplementation of anakinra is the drug of choice, with a very high response rate and achieving remission [31].

Deficiency of IL-36 receptor antagonist (DITRA)

DITRA is a rare autosomal recessive condition associated with a loss-of-function mutation of the IL36RN gene. It is characterized by recurrent fever episodes, pustular psoriasis, and sometimes life-threatening organ involvement. Topical treatments include topical corticosteroids, topical retinoids, emollients, moisturizers, and an adjunct to systemic treatment for cutaneous manifestations. Systemic treatment with corticosteroids, methotrexate, and cyclosporine is helpful for both systemic and cutaneous features. Secukinumab (an IL-17A inhibitor), ustekinumab (an IL12/23 blocking agent), and anti-TNF agents (infliximab, adalimumab) have a high success rate in DITRA. Overall, the results of anti-IL-1 agents are not encouraging. Spesolimab, an anti-IL-36 receptor blocker approved by the FDA for generalized pustular psoriasis, has shown positive results in a phase II trial in DITRA disorder [32].

Deficiency of adenosine deaminase 2 (DADA2)

DADA2 is inherited as an autosomal recessive pattern and associated with a mutation of the CECR1 gene. Clinical features resemble polyarteritis nodosa but are ANCA-negative. Low IgM levels are also observed in this disorder. DADA2 has four phenotypes: inflammatory and/or vasculitic, hematologic, immunodeficient, and presymptomatic. Vasculitis can cause ischemic or hemorrhagic stroke, and cutaneous or systemic polyarteritis nodosa. Hematologic abnormalities like anemia, leukopenia, thrombocytopenia, pancytopenia, and lymphoproliferative disorders are reported in some patients. Hypogammaglobulinemia may correlate with inflammatory disease activity. DADA2 Consensus Committee recommends a TNF inhibitor (etanercept) as the treatment of choice in patients with vasculitis, stroke, and systemic inflammation. Prophylactic antibiotics may be considered in children with hypogammaglobulinemia and recurrent infection. Allogenic stem cell transplantation is advised in patients with bone marrow failure or refractory cytopenia [33].

Cyclic neutropenia

Cyclic neutropenia is an autosomal dominant condition caused by a mutation of the ELANE gene. The episodes are characterized by recurrent febrile episodes lasting two to five weeks and associated with severe neutropenia. Some of the neutropenic attacks may be related to infections. Infection should be treated aggressively with appropriate antimicrobials. Granulocyte-colony stimulating factor (G-CSF) is effective during episodes for increasing neutrophil counts. Hematopoietic stem cell transplantation can permanently cure the condition and is advised in patients refractory to G-CSF therapy [34].

STING-associated vasculopathy with onset in infancy (SAVI)

SAVI is an interferonopathy caused by a mutation of the STING1 gene. It is characterized by neonatal onset interstitial lung disease (ILD) and cutaneous vasculitis involving the acrofacial region [35]. Treatment is challenging, and the outcome is usually poor with systemic involvement and early ILD. Corticosteroids, immunosuppressants, and biologicals have been tried with partial improvement. Janus kinase inhibitors tofacitinib, ruxolitinib, and baricitinib show promise in many cases [36].

Proteasome-associated autoinflammatory syndromes (PRAAS)

It occurs because of a defect in immunoproteasome PSMB8. It consists of three distinct clinical syndromes, namely, chronic atypical neutrophilic dermatosis with lipodystrophy and elevated temperature (CANDLE), Nakajo-Nishimura syndrome, and joint contractures, muscle atrophy, microcytic anemia, and panniculitis-induced childhood-onset lipodystrophy (JMP). Corticosteroids are helpful during acute episodes. Rebound symptoms occur during the tapering of corticosteroids. Response to anti-TNF agents, IL-1, and IL-6 inhibitors has been inconsistent and variable [37].

Disease monitoring

Monitoring systemic inflammation should be done in all diseases, which includes biomarkers of inflammation. Complete hemogram, differential count, erythrocyte sedimentation rate, CRP, and, when possible, serum amyloid A protein and S-100 protein should be done at each visit. Urine examination for proteinuria should be done every six to 12 months. Growth, bone mineral density, and sexual development should be assessed on each visit. Disease-specific symptoms and patient outcomes should be monitored on each visit. Disease-specific organ involvement and damage testing should be done at frequent intervals, such as urine analysis, hearing screening, ophthalmology screening, MRI/lumbar puncture, and other investigations as required [38]. Hematologic and biochemical parameters like complete hemogram, liver function test, and kidney function tests should be checked routinely to review drug-associated adverse events [39]. It is clear from the above discussion that biologics have clear advantages over conventional drugs in the management of most of the periodic fever syndromes. However, the risk of adverse effects following biologics is also substantial. Baseline complete hemogram, erythrocyte sedimentation rate (ESR), CRP, liver function test, and renal function test are usually ordered. Screening for hepatitis B, hepatitis C, and HIV should be done. Vaccination status should be enquired with special reference to live vaccines, and it is desirable to complete the course of vaccines if possible. The most important concern with biologics is reactivation of tuberculosis (TB), which is primarily observed with anti-TNF agents than non-TNF biologics [40]. In countries where TB prevalence is high, it is very important to rule out latent tubercular infection (LTBI) or active TB prior to initiation of biologics therapy. Achievement of clinical remission should be the primary target of therapy, and response to therapy should be evaluated by a validated autoinflammatory disease activity tool (Table 1).

**Table 1:** Brief summary of common periodic fever syndromes. FMF: familial Mediterranean fever; TRAPS: tumor necrosis factor receptor-associated periodic syndrome; CAPS: cryopyrin-associated periodic fever syndromes; FCAS: familial cold autoinflammatory syndrome; MWS: Muckle-Wells syndrome; NOMID: neonatal-onset multisystem inflammatory disease; HIDS: hyper IgD syndrome; PFAPA: periodic fever, aphthous stomatitis, pharyngitis, and adenitis syndrome; PAPA: pyogenic arthritis, pyoderma gangrenosum, and acne syndrome; DIRA: deficiency of the interleukin-1 receptor antagonist; DITRA: deficiency of the interleukin-36 receptor antagonist; DADA2: deficiency of adenosine deaminase 2; SAVI: STING-associated vasculopathy with onset in infancy; PRASS: proteasome-associated autoinflammatory syndromes; NSAIDs: nonsteroidal anti-inflammatory drugs; IVIG: intravenous immunoglobulin; AD: autosomal dominant; AR: autosomal recessive; G-CSF: granulocyte-colony stimulating factor; TNF: tumor necrosis factor.

Disease	Inheritance	Gene involved	Clinical features	Treatment options
FMF	AR	MEFV	Fever lasting 1-3 days, serositis, skin, and joint involvement	Colchicine, anakinra, canakinumab, rilonacept
TRAPS	AD	TNFRSF1A	Fever duration 1-4 weeks, eye and skin changes	Corticosteroids, etanercept, anakinra, canakinumab
CAPS (FCAS/MWS/NOMID)	AD	NLRP3	Fever, urticarial skin rash, joint involvement	Anakinra, rilonacept, canakinumab
HIDS	AR	MVK	Fever, skin rash, joint pain, hepatosplenomegaly, lymphadenopathy	NSAIDs, colchicine, glucocorticoids, IVIG, canakinumab, etanercept, tocilizumab
PFAPA	-	-	Fever lasting 5 days, cervical adenitis, pharyngitis, tonsillitis, aphthous ulcer	NSAIDs, colchicine, cimetidine, ketotifen, adenotonsillectomy, anakinra
PAPA	AD	PSTPIP1	Sterile erosive arthritis, ulcerative skin lesion	Etanercept, anakinra, methotrexate
DIRA	AR	ILRN1	Fever, sterile osteolytic lesion, skin pustules	Anakinra
DITRA	AR	IL36RN	Fever, pustular psoriasis, systemic involvement	Topical corticosteroids, retinoids, and emollients. Systemic: corticosteroid, methotrexate, cyclosporine, apremilast, secukinumab, ustekinumab, infliximab, adalimumab
DADA2	AR	CECR1	Vasculitic, inflammatory changes like polyarteritis nodosa	Etanercept, prophylactic antibiotics, and allogenic stem cell transplantation
Cyclic neutropenia	AD	ELANE	Neutropenia, recurrent infection	Antibiotics, G-CSF, hematopoietic stem cell transplantation
SAVI	AD	STING1	Neonatal onset interstitial lung disease, cutaneous vasculitis	Corticosteroid, immunosuppressant, tofacitinib, ruxolitinib, baricitinib
PRASS		PSMB8	Chronic neutrophilic dermatosis, lipodystrophy, anemia, joint contracture	Corticosteroids, anti-TNF agents, IL-1, and IL-6 inhibitors

## Conclusions

Hereditary periodic fever syndromes are rare autoinflammatory diseases associated with characteristic clinical manifestations. Most diseases are monogenic, start in childhood, and need genetic analysis to confirm diagnosis. IL-1 is the most common cytokine in most diseases implicated in fever pathogenesis and other systemic manifestations. Treatment includes colchicine, corticosteroids, immunosuppressants, and biologics, which are disease-specific. Monitoring disease activity, biological screening for serious infections, and drug toxicity are essential to avoid untoward complications and improve outcomes. There are research gaps on the standard therapy in many periodic fever syndromes, the duration of treatment, and long-term outcomes. With the advances in molecular genetics and better characterization of pathogenesis, the treatment of these disorders may see changes in the future with the addition of novel therapeutics.
